# Role of imaging in female infertility [Dr. K.M. Rai Memorial Oration Award]

**DOI:** 10.4103/0971-3026.69347

**Published:** 2010-08

**Authors:** Rajul Rastogi

**Affiliations:** Yash Diagnostic Center, Yash Hospital & Research Center, Civil Lines, Kanth Road, Moradabad (UP), India

**Keywords:** Infertility, magnetic resonance imaging, ultrasonography

## Abstract

Infertility in females is multifactorial in origin. Though hysterolaparoscopy is the gold-standard investigation, USG is usually the first-line investigation. MRI has expanded the usefulness of imaging in female infertility. This pictorial essay reviews the role of imaging in the evaluation of female infertility.

## Introduction

Infertility is defined as failure to conceive a desired pregnancy after 12 months of unprotected intercourse.[1] Approximately 10% of married couples are infertile. Males and females are equally affected. The causes of female infertility can be broadly categorized into the following:

Uterine causes – Congenital anomalies, infections, uterine synechiae, focal lesions, intrauterine scar, cervical stenosis, reduced uterine perfusion, and alterations in endometrial thickness and vascularity.

Ovarian causes – Follicular and ovulation abnormalities, stromal vascularity, and endometriosis.

Tubal causes – Infections, obstruction.

## Diagnostic Armamentarium and its Role

### Ultrasonography (USG) (mainly transvaginal/endovaginal)

It is the first-line investigation and can be coupled with color Doppler and 3D/4D scans.[2,3] It is readily available, inexpensive, noninvasive, radiation-free, relatively less time consuming, and easily repeatable. Limitations include subjective errors, limited field of view, interference by obesity or by gaseous bowel loops, suboptimal visualization of fallopian tubes and broad ligament, failure to delineate small ovaries, and inability to obtain images in the surgical plane.

USG helps in determining the morphology of the uterus and ovaries, uterine and ovarian perfusion, and endometrial thickness, volume, and vascularity. It detects pathological lesions, including tubal lesions and abnormalities of follicular maturation and ovulation. Tubal patency can be confirmed through sonosalpingography. USG can guide oocyte retrieval and embryo transfer in *in vitro* fertilization procedures and drainage of pelvic collections or cystic lesions.

### Magnetic resonance imaging (MRI)

It is best for delineating the morphology and orientation of pelvic structures. Though it is noninvasive and radiation-free, it has limited availability and high cost, and hence cannot be repeated easily.[2,3] Longer examination time, failure to delineate sub-centimeter uterine lesions, and inability to characterize endometriomas at some stages are other limitations. MRI is contraindicated in patients with cardiac pacemakers and cochlear implants.

MRI also detects pathological lesions, including tubal lesions and pituitary adenoma. It helps in predicting the prognosis in conservatively treated cases of leiomyoma, adenomyosis, and endometriosis.

### X-ray hysterosalpingography (HSG)

It is a less-preferred procedure used to visualize the uterine cavity and confirm tubal patency. It involves ionizing radiation and carries the risk of infection and injury.

### Sonohysterosalpingography (Sono-HSG)

It involves airless, sterile, saline infusion through a soft plastic catheter in the cervix with simultaneous endovaginal USG. It allows excellent visualisation of the endometrial cavity and its lining. The procedure can also confirm tubal patency by demonstrating spillage of saline from a distended tube into the pelvic cavity.

## Causes of Female Infertility

### Polycystic ovarian syndrome (PCOD) [Figure 1]

This is characterized by a combination of multiple clinical manifestations (i.e., hirsutism, menstrual disturbances, anovulatory cycles, and infertility) and hormonal imbalance (an abnormal luteinizing hormone / follicular stimulating hormone (LH/FSH) ratio and excessive androgen secretion).

**Figure 1 (A-D):**
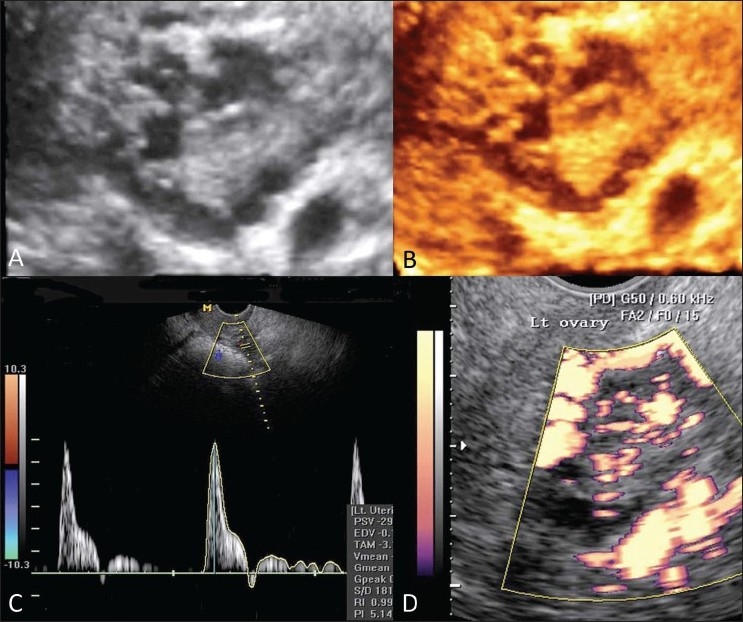
Polycystic ovary. Three-dimensional gray-scale (A) and color (B) images show a polycystic ovary. Spectral Doppler (C) and power Doppler (D) images show reduction in diastolic flow, reverse diastolic component, and increased stromal vascularity in polycystic ovary

USG characteristically reveals rounded ovaries with normal (30% case) or increased volume, multiple peripheral, sub-centimeter follicles (at least 15) with no dominant follicle (‘string-of-pearls’ appearance), thickened walls, and an echogenic and vascular stroma. Bulky and thick-walled ovaries with multiple, peripheral, sub-centimeter T2-hyperintense cysts and hypointense stroma are characteristic findings on MRI.[4]

### Pituitary adenoma

MRI is the modality of choice for detecting pituitary adenoma. Microadenoma (<1 cm) is usually hypointense to the normal pituitary on T1W images. Convex pituitary contour and deviations of the pituitary stalk are indirect signs. Dynamic postcontrast MRI reveals strong enhancement of the normal pituitary and its stalk in the early phase in contrast to the faint enhancement of a microadenoma.[5] Macroadenomas (>1 cm) may compress/invade surrounding structures, including the optic chiasm, cavernous sinus, and bony sella.

### Tubal diseases [Figure 2]

These mainly include destruction or obstruction and peritubal adhesions.

**Figure 2 (A-C):**
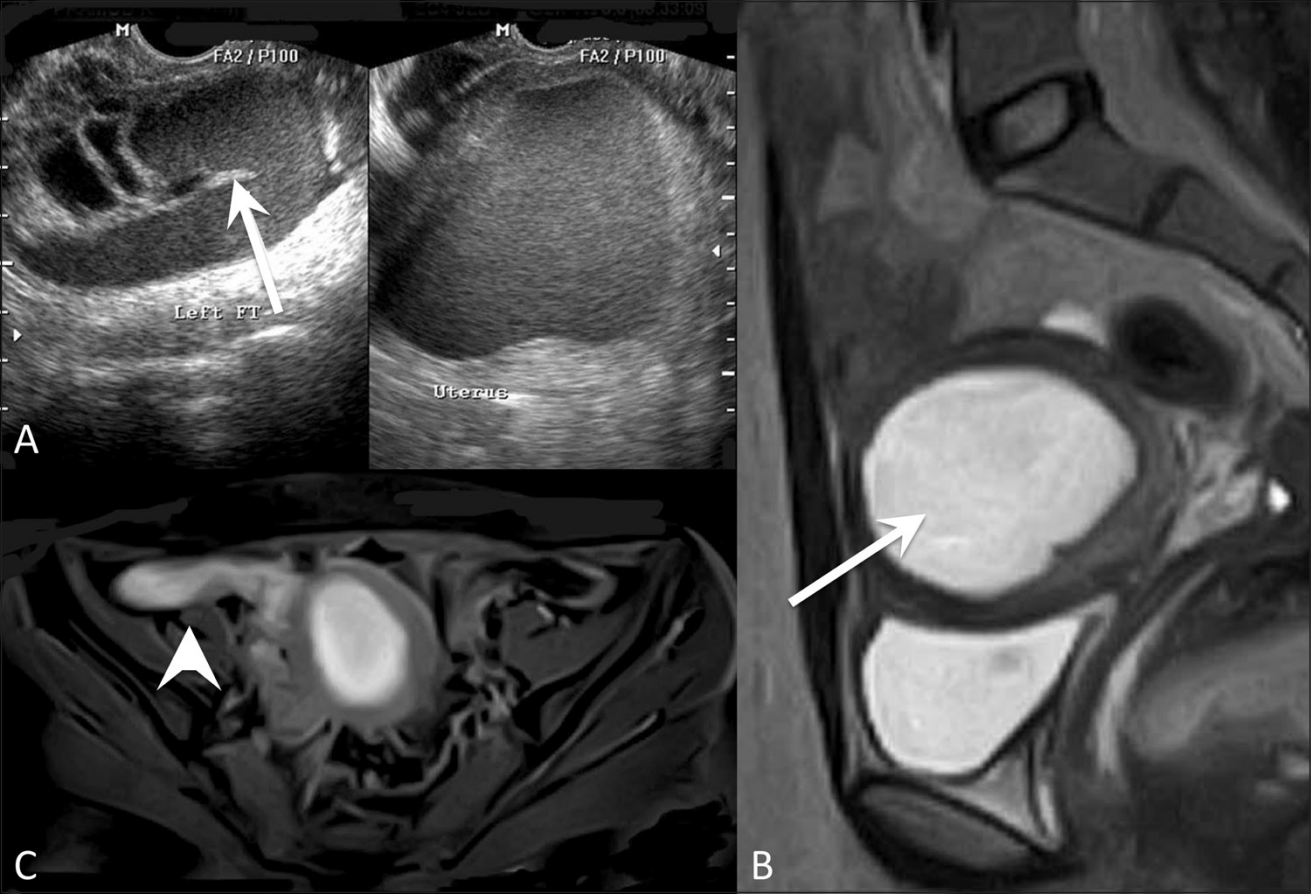
Hematometra with hematosalpinx. Transvaginal USG (A), sagittal T2W (B) and axial fat-suppressed T1W (C) MRI images show hematometra (arrow) with hematosalpinx (arrowhead) on the right side. Note incomplete septae in tube (white arrow in A)

HSG is useful for assessing tubal patency. Recently, MRI-based HSG has also been introduced.[6] MRI is superior to USG for studying the tubes. Dilated tubes appear as fluid-filled, tortuous, sausage-shaped masses adjacent to the uterus with incomplete septae appearing as hyperechoic mural nodules (beads on string sign) and short linear projections (cogwheel appearance). The presence of partially effaced longitudinal folds inside the masses is specific for fallopian tubes on MRI.[7] The presence of a normal-appearing ipsilateral ovary is a clue to the presence of a tubal mass.

### Pelvic inflammatory disease (PID) [Figures 3–5]

PID is a common cause of infertility and can manifest as pelvic collections, tubo-ovarian collections, uterine or broad ligament infection.

**Figure 3 (A,B):**
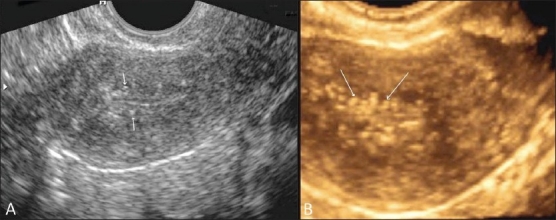
Pelvic inflammatory disease. Transvaginal 2D gray-scale (A) and 3D color (B) USG images show subendometrial calcification (white arrows)

**Figure 4 (A,B):**
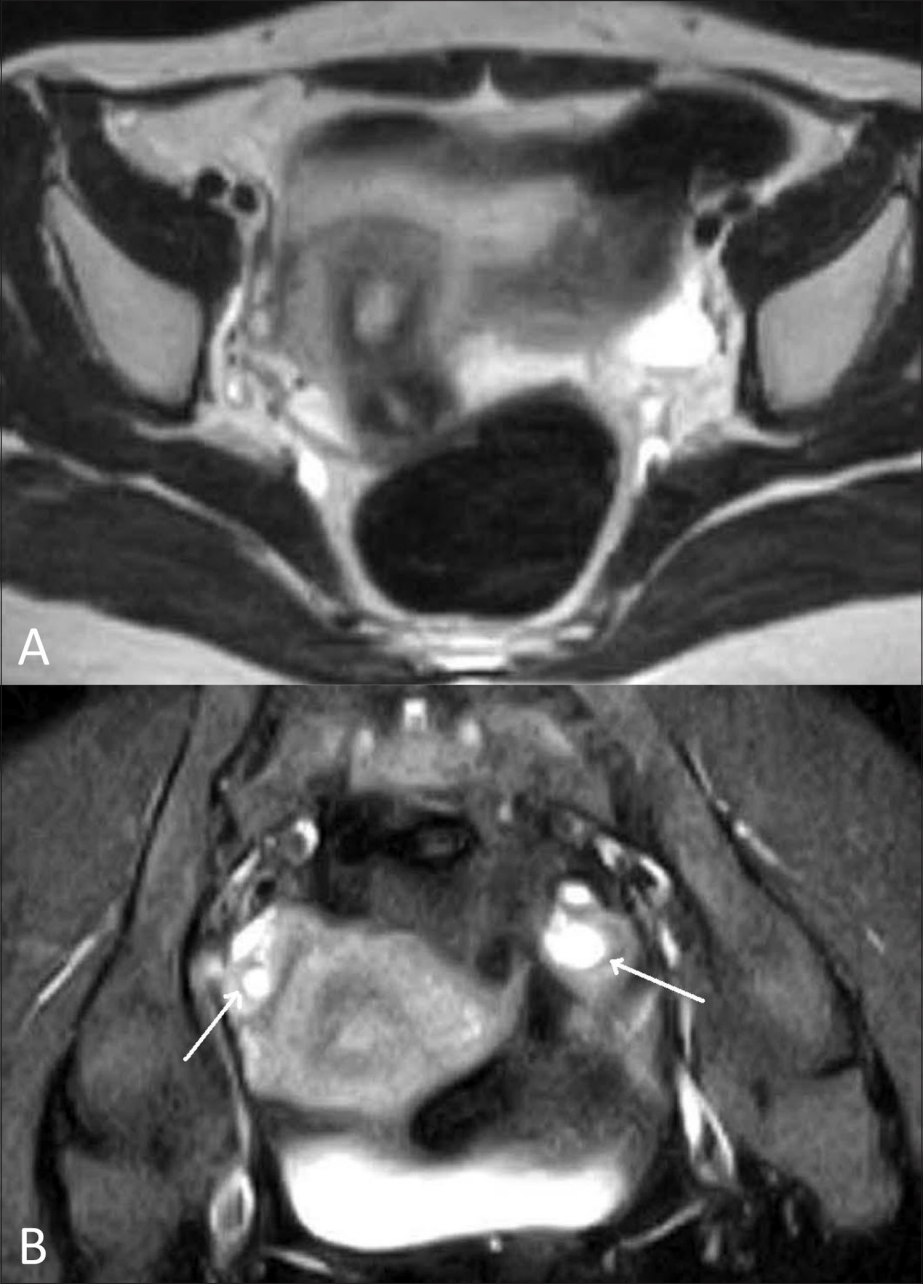
Pelvic inflammatory disease. Axial T2W (A) and oblique coronal fat-suppressed T2W (B) MRI images show pelvic inflammatory disease with resorbed tubes, an infected broad ligament, and thickwalled ovaries (white arrows)

**Figure 5 (A,B):**
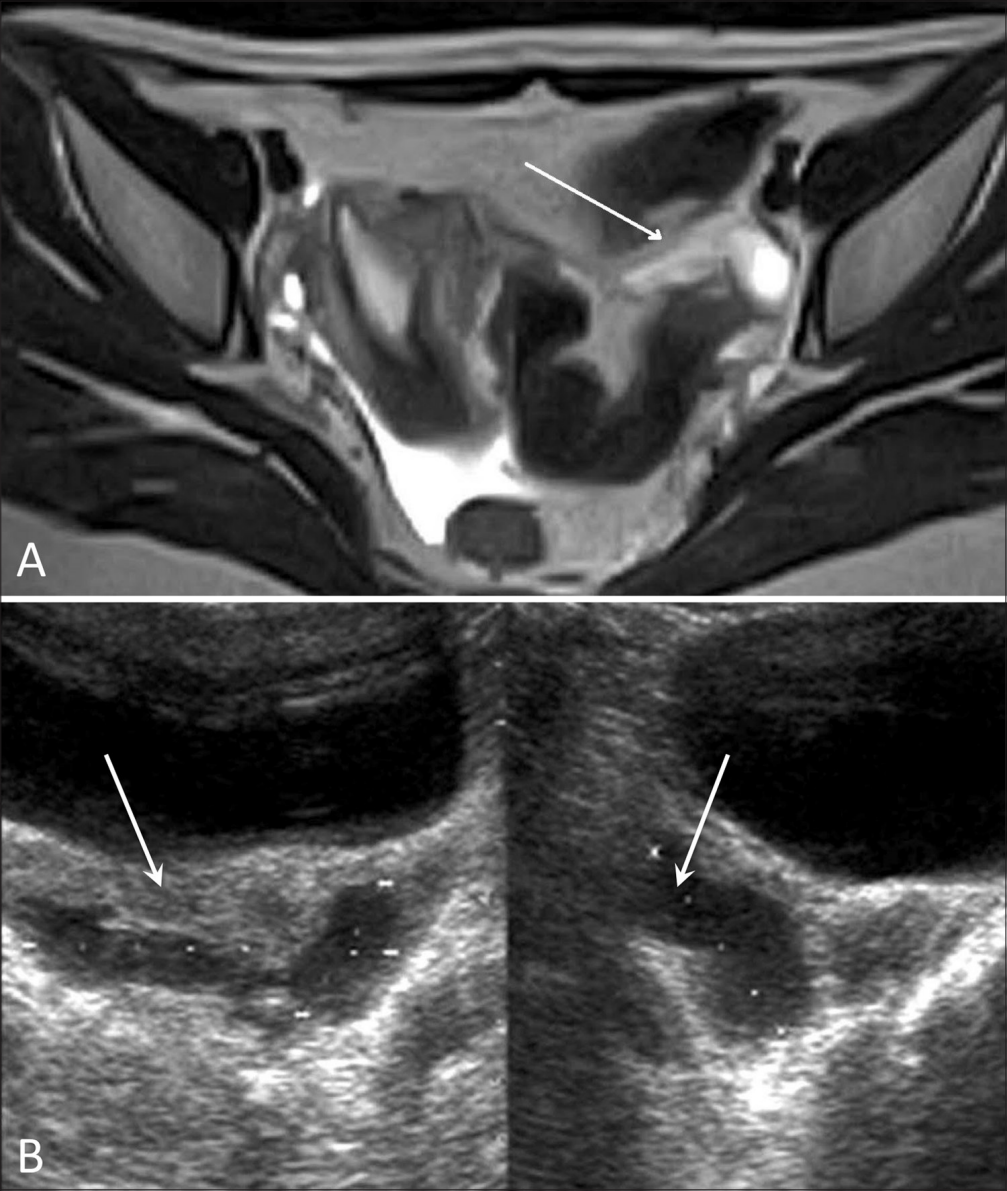
Pelvic inflammatory disease. Axial T2W MRI (A) and transabdominal USG (B) images show pelvic inflammatory disease with a digested/damaged left tube and mild collection in POD (white arrows)

USG and MRI are equally sensitive in detecting tubo-ovarian collections. The presence of peripheral vascularity of high-resistance type on color Doppler USG is suggestive of an infective mass. The presence of a high-signal-intensity inner rim on T1W images and enhancement on postcontrast images are helpful signs.[8] MRI is superior to USG for revealing an infected uterus and broad ligament which appear hyperintense on T2W images.

Other signs of PID include probe tenderness, thickening of the tubes (mural thickness more than 5 mm) and tubo-ovarian masses (tube and ovary identifiable but inseparable).

### Endometriosis [Figure 6]

This condition mostly involves the ovaries but can secondarily involve other pelvic structures. USG is the preferred technique and shows a typical endometrioma located in the ovary as a well-defined cystic lesion with homogeneous low-level internal echoes (chocolate cyst) (more than 95%). It may also appear as an anechoic cyst, cystic mass with fluid-debris level or as a solid-appearing mass with or without thick septae. The presence of hyperechoic wall foci is characteristic on USG. MRI is more sensitive in detecting an endometrioma which appears hyperintense on T1W images and hypo- to hyperintense on T2W images. Fat-suppressed T1W images are very useful for detecting peritoneal implants.[9]

**Figure 6 (A-D):**
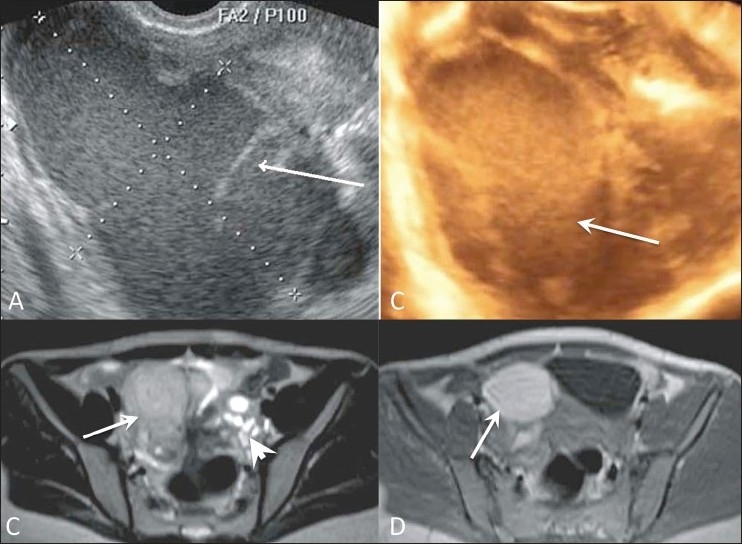
Endometrioma. Transvaginal gray-scale (A), 3D color USG (B) USG, axial T2W (C) and T1W (D) MRI images show an endometrioma (arrows) on the right side and a polycystic ovary (arrowhead) on the left side in two different patients. Note the thick septae in the endometrioma (arrow in A)

The tubes may be involved in the form of a hematosalpinx or with peritubal adhesions, while uterine involvement appears as adenomyosis. Adhesions are seen on MRI as hypointense strands within the adjacent fat, obscuring adjacent interfaces. A posteriorly displaced uterus, kissing ovaries (both ovaries lying in the pouch of Douglas (POD) inseparable from each other), elevated posterior vaginal fornix, angulated small bowel loops, hydro-/hematosalpinx, and multilocular fluid collections are indirect indicators of pelvic adhesions.[10]

### Leiomyoma [Figure 7]

It infrequently causes infertility by interfering with transportation of sperms or implantation due to distortions of the uterine contour and cavity.[11] Endovaginal USG is as sensitive as MRI in the detection of leiomyoma. MRI however is superior in the preoperative evaluation of the site, number, and size of leiomyomas.[12]

**Figure 7:**
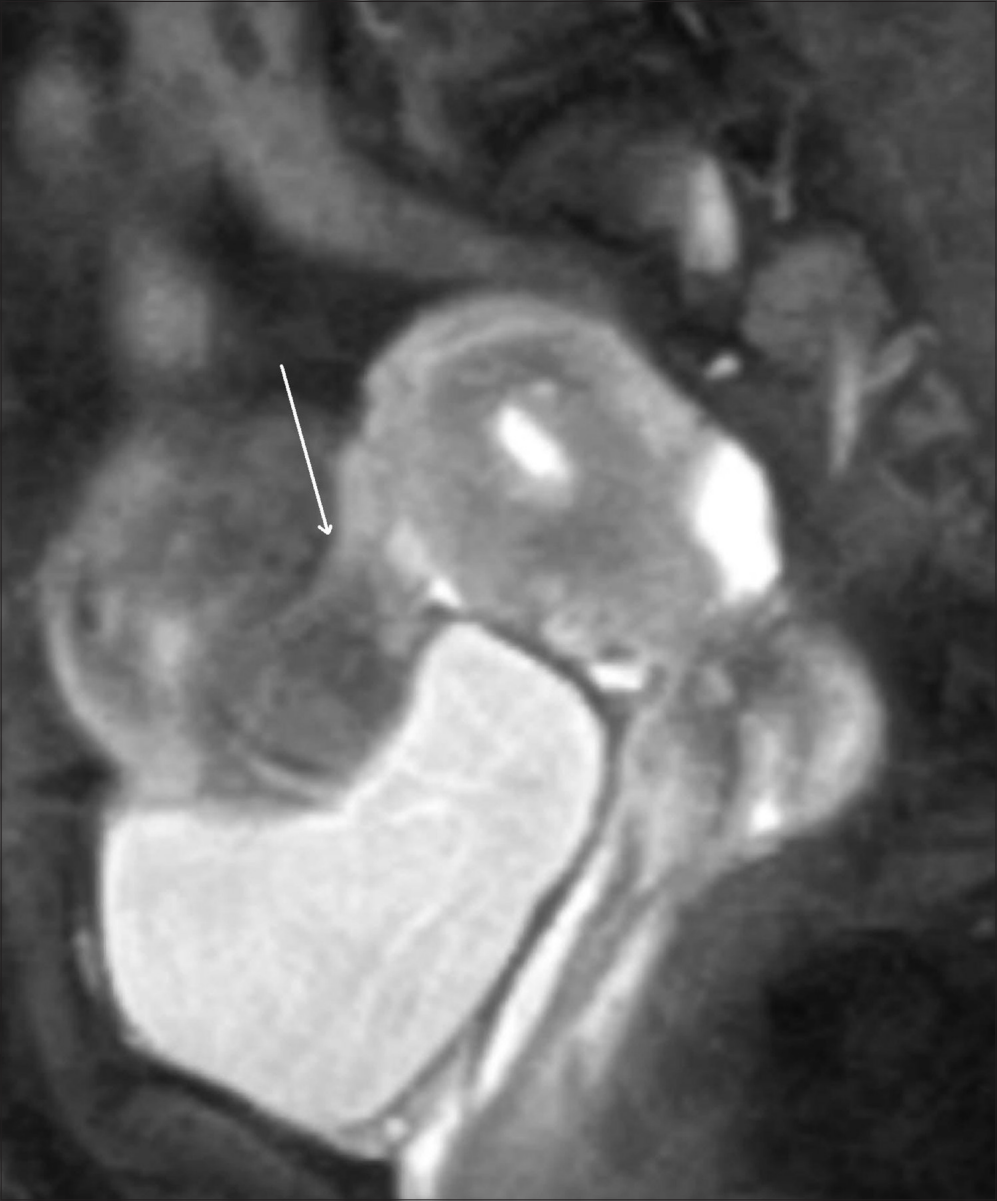
Subserosal fibroid. Sagittal fat-suppressed T2W MRI image shows a pedunculated subserosal fibroid along the anterior wall (white arrow shows the stalk)

A typical leiomyoma on USG appears as a well-defined, hypoechoic to heteroechoic solid lesion with variable posterior acoustic shadowing located in a submucosal, intramural, or subserosal location. Color Doppler reveals peripheral vascularity of mild to moderate resistance, differentiating it from an adenomyoma which reveals moderate central and peripheral vascularity of relatively low resistance. Leiomyoma appears hypointense to myometrium on both T1W and T2W images.

MRI can monitor post-treatment changes and recurrences in patients treated with uterus-conserving methods of treatment.[13]

### Adenomyosis [Figure 8]

It uncommonly causes infertility possibly by reducing uterine/endometrial receptivity. Both endovaginal USG and MRI are equally sensitive for making a diagnosis.

**Figure 8 (A-C):**
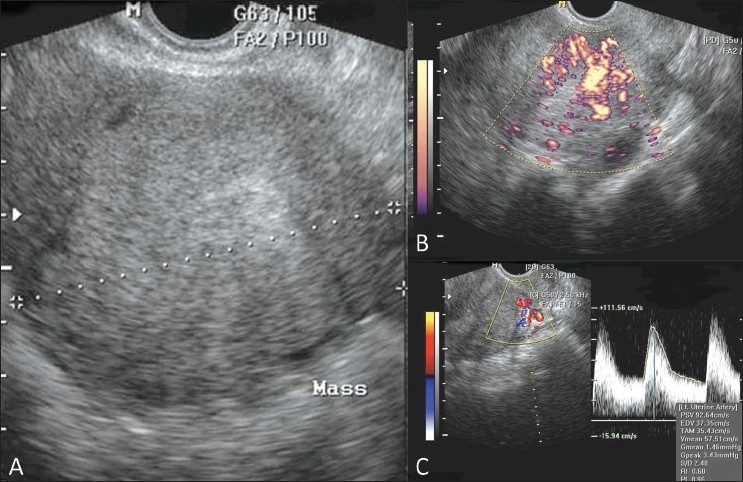
Adenomyoma. Transvaginal 2D gray-scale (A), power Doppler (B) and triplex Doppler (C) USG images show a uterine adenomyoma

USG findings include a diffusely enlarged or globular uterus, asymmetric walls (>2.5 cm), ill-defined areas or diffusely altered uterine echogenicity, myometrial or subendometrial cysts, indistinct endometrial-myometrial interface, subendometrial echogenic nodules or strands with surrounding hypoechoic myometrium, and undulating outer margin of endometrium. MRI findings include hypointense masses with poorly defined margins on both T1W and T2W images; focal or diffuse, symmetric or asymmetric, widening of the junctional zone (>12 mm) of the uterus; presence of hyperintense foci representing ectopic endometrium on T2W images.[14] MRI can be used for monitoring patients undergoing conservative gonadotropin-releasing hormone (GnRH) analogue therapy.[15]

### Uterine anomalies (Müllerian duct anomalies) [Figures 9 and 10]

These are considered as causes of infertility when all other causes have been excluded. Multiplanar MRI is diagnostic. These are classified according to the American Fertility Society criteria as follows[16]:

- Class I or uterine hypoplasia or agenesis
- Class II or unicornuate uterus: A banana-shaped uterus with a single fallopian tube. A rudimentary horn (communicating or noncommunicating) may be present
- Class III or uterus didelphys: Two complete uteruses, each with its own cervix. A sagittal vaginal septum is seen in the majority of cases.
- Class IV or bicornuate uterus: Two uterine cavities with one cervix. MRI shows widely separated uterine horns with an intercornual distance of >4 cm and concavity of the fundal contour or an external fundal cleft of >1 cm in depth.[17]
- Class V or septate uterus: A fibrous septum is seen that appears hypointense on T2W images while the muscular septum appears intermediate in intensity. MRI criteria includes a convex or flat external fundal contour or external fundal cleft of <1 cm in depth.[18]
- Class VI or arcuate uterus: It is a normal variant and is characterized by an external convex contour of the fundus with fundal endometrial indentation.
- Class VII or diethylbestrol-induced: Exposure to this synthetic estrogen antenatally can result in a T-shaped, hypoplastic, and constricted uterus.

**Figure 9 (A,B):**
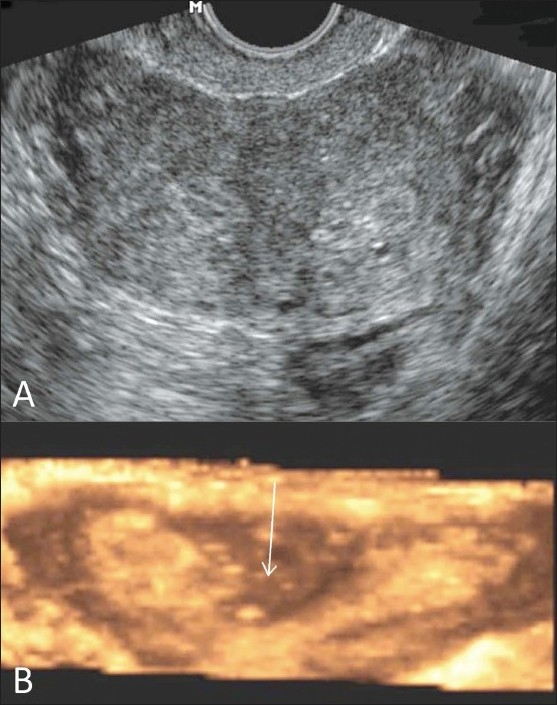
Uterus subseptus. Transvaginal 2D gray-scale (A) and 3D color coronal (B) USG images show a uterus subseptus (white arrow shows the incomplete septum)

**Figure 10 (A,B):**
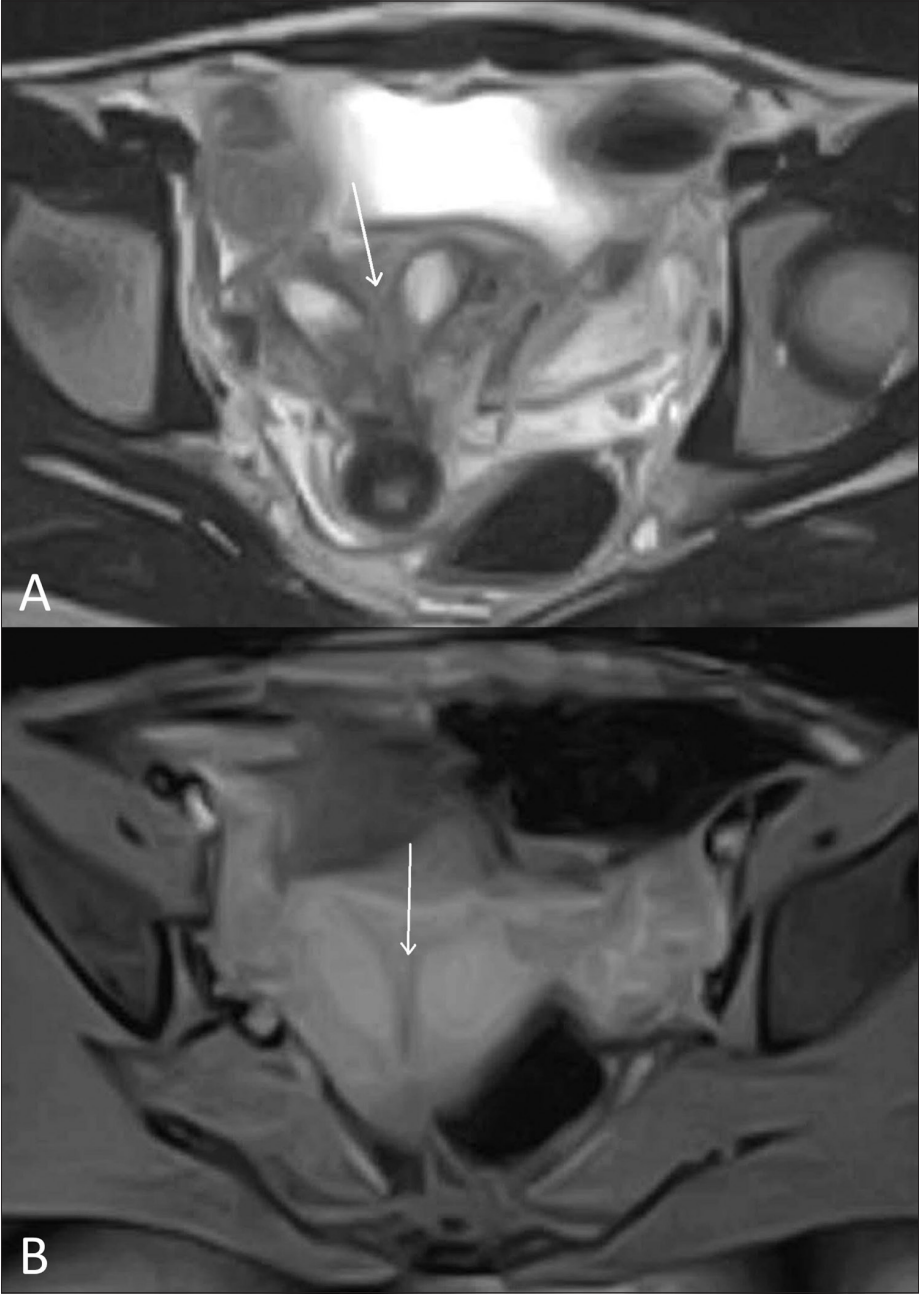
Uterus subseptus. Axial T2W (A) and fat-suppressed T1W (B) MRI images show a uterus subseptus (white arrows show incomplete septum)

## Conclusion

USG is the investigation of first choice in infertile females, as it is highly accurate in determining common causes of infertility.

MRI should be used as a second-line tool in patients with complex clinical manifestations with normal USG; equivocal, atypical, or multiple USG findings; or when a particular question cannot be answered on USG. It serves as a useful adjunct to diagnostic hysterolaparoscopy, especially in patients with tubal diseases and endometriosis.
